# Gastrointestinal stromal tumor causing ileo-ileal intussusception in an adult patient a rare presentation with review of literature

**DOI:** 10.4314/pamj.v8i1.71086

**Published:** 2011-03-20

**Authors:** Amit Gupta, Sweety Gupta, Ashutosh Tandon, Mrinalini Kotru, Sunil Kumar

**Affiliations:** 1 Department of Surgery; 2 Department of Oncology; 3 and Department of Pathology University College of Medical Sciences and associated Guru Teg Bahadur Hospital, New Delhi, India

**Keywords:** Gastrointestinal stromal tumor, Ileo-ileal intussusception, Acute small bowel obstruction

## Abstract

Gastrointestinal stromal tumors (GIST) are mesenchymal tumors occurring anywhere along the gastrointestinal tract and are believed to originate from the interstitial cells of Cajal. They commonly arise in the stomach or small intestine. The usual growth pattern is exophytic invading adjacent organs or perforation into the peritoneal cavity which may result in bleeding or obstructive symptoms. Intussusception and obstruction is a very uncommon presentation of these lesions because of their tendency to grow in an extraluminal fashion. We report an unusual case of 59 yrs old man presenting with acute small bowel obstruction, which on exploration was found to be due to ileo-ileal intussusception and the lead point of intussusception was a tumor, which was histologically diagnosed as GIST

## Introduction

Gastrointestinal stromal tumors (GIST) are mesenchymal tumors occurring anywhere along the gastrointestinal tract and are believed to originate from the interstitial cells of Cajal. They commonly arise in the stomach or small intestine. The usual growth pattern is exophytic invading adjacent organs or perforation into the peritoneal cavity which may result in bleeding or obstructive symptoms. Intussusception and obstruction is a very uncommon presentation of these lesions because of their tendency to grow in an extraluminal fashion. We report an unusual case of 59 yrs old man presenting with acute small bowel obstruction, which on exploration was found to be due to ileo-ileal intussusception and the lead point of intussusception was a tumor, which was histologically diagnosed as GIST.

## Patient and case report

**Figure 1:**
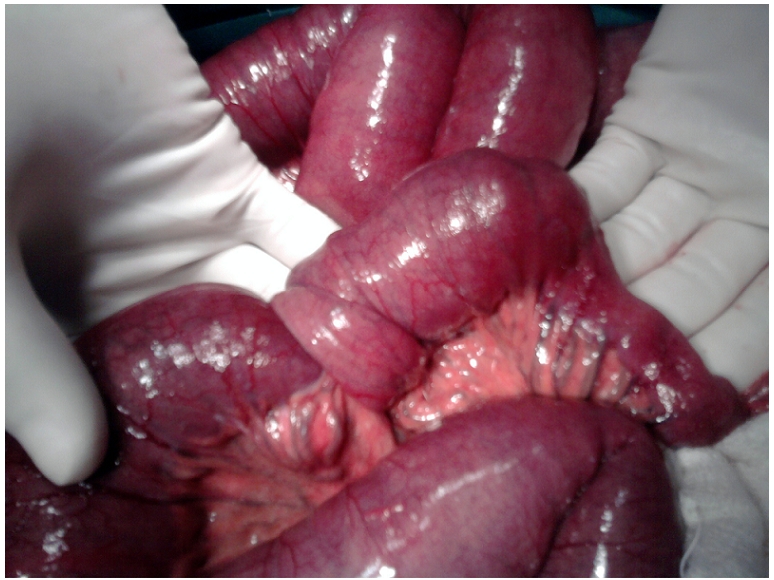
Photograph showing intramural mass arising from the wall of ileum (making the lead point of intussusception)

**Figure 2:**
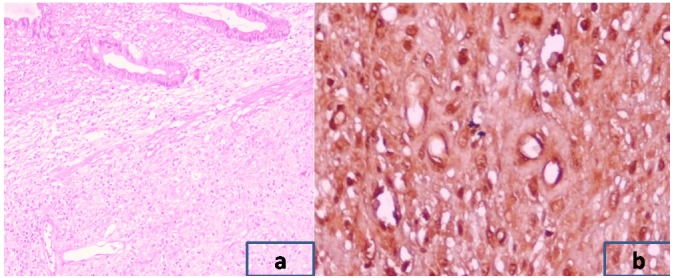
(a) Intestinal mucosa with tumor in the submucosa (H&E,X40) (b) Immunohistochemical staining with CD117 (X400)

A 59 yrs. old man was admitted in the surgical emergency with 3 days history of abdominal pain, distension and multiple episodes of bilious vomiting. He also had complaint of constipation lasting for the previous 2 days. There was no significant past medical history. On physical examination, he had tachycardia and hypotension; temperature was normal. The abdomen was distended with visible peristalsis. There was generalized tenderness on deep palpation. No palpable mass was identified. Bowel sounds were exaggerated. Digital rectal examination was unremarkable. Laboratory investigations showed leukocytosis and raised blood urea (52mg/dl) but serum creatinine was within normal range. Liver function tests were normal. Plain x-ray abdomen showed multiple air fluid levels. Ultrasonography abdomen showed dilated bowel loops, with no evidence of any mass or free fluid in the peritoneal cavity. With all these findings suggestive of acute intestinal obstruction, patient was planned for urgent exploratory laparotomy. Intra operatively ileoileal intussusception was present two and half feet proximal to ileo-ceacal junction. The bowel proximal to this area was dilated. There was an intramural mass arising from the wall of ileum making the lead point of intussusceptions (Figure 1). There were no signs of bowel gangrene. Mesenteric lymph nodes were enlarged. There was no free fluid and inter bowel adhesions. Resection of the ileal segment bearing the mass with end-to-end ileoileal anastomosis was done along with excision biopsy which was taken from the mesenteric lymph node. Histopathological diagnosis turned out to be gastrointestinal stromal tumor (Figure 2), which was positive for c-kit and CD 117. Mesenteric lymph node biopsy showed chronic non specific inflammation. Patient recovered well in postoperative period and was discharged on 8^th^ postoperative day. Patient was referred to Oncology clinic for chemotherapy.

## Discussion

Intussusception, defined as the telescoping of a segment of the gastrointestinal tract into an adjacent one, is extremely rare in the stomach, but more common in the small intestine, ileocecal junction and colon. Intussusception is correctly diagnosed preoperatively in only one-third of cases. An accurate diagnosis of intussusceptions should include a good history, a thorough physical examination, radiography, CT, MRI and enteroclysis or even an endoscopic ultrasound or capsule endoscopy [1]. Ultrasound is useful in confirming obstruction and may sometimes identify the cause. Few reports are available which shows that Ultrasonography should be the initial diagnostic investigation in which they have found a typical multilayered bowel wall (“target-like sign” or “bull´s eye sign”) [2].

It has been recently reported that CT and MRI offer great help in establishing the preoperative diagnosis of intussusception. In our case, ultrasound was not diagnostic of intussusception and CT could not be done because of unstable vitals of the patient. In contrast to childhood where intussusception is idiopathic in 90% of cases and the basic underlying cause of intussusception is the hypertrophy of Payer’s patches activity, adult intussusception has a definable pathologic lesion in over 90% of cases, with neoplasms considered to be the cause in 65% of them [3, 4].

Any intraluminal lesion, especially polyps, which irritates and alters normal peristaltic activity, is able to trigger an intraluminal invagination finally causing an intussusception. Subsequent peristaltic bowel activity produces an area of sequence constriction and relaxation, thus telescoping the leading point through the distal bowel lumen. The malignancy is more likely to be located in the colon rather than in the small bowel. Less common etiologies of intussusception in adults include postoperative factors (adhesions, suture lines, etc.), polyps, Meckel’s disease, sprue, cecal duplication and intramural hematoma. The presentation of adult intussusception is usually subacute or chronic. Only up to 20% of all cases present with complete bowel obstruction and acute onset. In the literature, very few cases of small bowel intussusceptions from a stromal tumor in adults have been described [5, 6].

Gastrointestinal stromal tumors (GISTs) are a subset of mesenchymal tumors of varying differentiation. They are rare clinical entities, constitute less than 3% of all gastrointestinal malignant neoplasms and represent only 20% of small-bowel malignant neoplasms (excluding lymphoma) [7]. They are typically defined as a tumor whose behavior is driven by mutations in the Kit gene or PDGFRA gene, and may or may not stain positively for Kit. 95% of all GISTs are CD117-positive (other possible markers include CD34, DOG-1, desmin, vimentin and others). Other cells that show CD117 positivity are mast cells [8]. If the CD117 stain is negative and suspicion remains that the tumor is a GIST, the newer antibody DOG-1 (Discovered on GIST-1) can be used. Also sequencing of Kit and PDGFRA can be used to prove the diagnosis. Patients present with trouble in swallowing, gastrointestinal hemorrhage or metastases (mainly in the liver). Intestinal obstruction is rare, due to the tumor’s outward pattern of growth. Surgery is the primary therapeutic option with the goal being complete resection for non metastatic tumors. Lymph node metastases are rare and routine removal of lymph nodes is typically not necessary [9]. Until recently, GISTs were notorious for being resistant to chemotherapy, with a success rate of < 5%. The discovery of activating mutations in the tyrosine kinases kit and more recently platelet-derived growth factor receptor alpha has stimulated development of therapeutic agents targeting these moieties. Imatinib mesylate is a tyrosine kinase inhibitor, being routinely used routinely in the management of GIST. This oral agent is well tolerated and highly effective for patients with metastatic GIST [10]. Adjuvant treatment with imatinib following surgical resection of GIST tumors can significantly reduce the risk of disease recurrence (6% recurrence on imatinib vs. 17% without therapy at 12 months).

## Conclusion

GIST are a subset of mesenchymal tumors and represent the most common mesenchymal neoplasms of Gastrointestinal tract. The clinical presentation of GIST is erratic. Furthermore, only 70% of the patients are symptomatic, while 20% are asymptomatic and 10% are detected at autopsy. The symptoms and signs are not disease - specific and as a consequence, about 50% of GISTs have already metastases at the time of diagnosis. Only up to 20% of all cases present with complete bowel obstruction and acute onset. In the literature, very few cases of small bowel intussusceptions from a stromal tumor in adults have been described. We reported intussusceptions as an unusual presentation of GIST.

## Competing interests

The authors declare no competing interests.

## Authors’ contribution

All the authors have contributed to the management of the write up of the manuscript. All authors have read and approve the final version of the manuscript. Amit Gupta had operated the case and made the diagnosis. He was assisted in the operation by Ashutosh Tandon. Mrinalini Kotru has given the histopathological consultation.
